# Causal association between body mass index and temporomandibular disorders: a bidirectional two-sample Mendelian randomization analysis

**DOI:** 10.1186/s12903-023-03179-5

**Published:** 2023-07-18

**Authors:** Xin Chen, Zheng Cheng, Junyu Xu, Zhibai Zhao, Qianglin Jiang

**Affiliations:** 1 Department of Oral and Maxillofacial Surgery, Jiangyin People’s Hospital Affiliated to Nantong University, No.163, Shoushan Road, Jiangyin, 214400 Jiangsu Province China; 2 Department of Oral Mucosal Diseases, The Affiliated Stomatological Hospital of Nanjing Medical University, 136 Hanzhong Road, Nanjing, 210000 Jiangsu China

**Keywords:** Body mass index, Mendelian randomization analysis, Temporomandibular disorders, Temporomandibular joint disorders

## Abstract

**Background:**

Observational studies have shown that body mass index (BMI) is highly correlated with the occurrence of temporomandibular disorders (TMDs). However, these studies failed to present a causal relationship. Thus, we aimed to performed a Mendelian randomization (MR) study to investigate causality between BMI and TMDs.

**Methods:**

We performed a two-sample bidirectional MR analysis using large-scale genome-wide association studies (GWAS). Data were obtained from a large-scale BMI dataset (N = 322,154), TMDs dataset (N = 134,280). The causal effects were estimated with inverse-variance weighted (IVW) method, MR Egger, weighted median. Sensitivity analyses were implemented with Cochran’s Q test, MR-Egger intercept test, MR-PRESSO, leave-one-out analysis and the funnel plot.

**Results:**

In the forward MR analysis, a genetic prediction of low BMI was causally associated with a higher risk of TMDs (IVW OR: 0.575, 95% CI: 0.415–0.798, *p*: 0.001). Similar results were obtained using other complementary methods (MR Egger OR: 0.270, 95% CI: 0.104–0.698, *p*: 0.009; weighted median OR: 0.496, 95% CI: 0.298–0.826, *p*: 0.007). In the reverse MR results, TMDs was shown to have no significant effect on BMI (all *p* > 0.05). No pleiotropy and heterogeneity were detected in the bidirectional analysis (*p* > 0.05).

**Conclusion:**

A lower BMI might be causally associated with increased risk of TMDs, supporting the importance of weight control for the prevention of TMDs. Clinicians should pay more attention to the low-BMI patients among those seeking medical advice due to temporomandibular joint discomfort.

**Supplementary Information:**

The online version contains supplementary material available at 10.1186/s12903-023-03179-5.

## Background

Temporomandibular disorders (TMDs), characterized by preauricular pain, irregular jaw function and joint sounds, have marked and profound effects on daily life [1]. Epidemiological studies have shown that TMDs affect 10–15% adults [1] and its annual financial costs amount is more than 100 billion dollars in the United States [2]. The etiology of TMDs is multifactorial but still unclear. According to the Diagnostic Criteria for Temporomandibular Disorders (DC/TMD), the diagnosis of TMDs involves Axis I for the clinical examination and Axis II for the pain-related disability [3]. Myogenic TMDs often present with more severe pain than arthrogenous and mixed TMDs [4]. Traditional conservative management schemes include patient education, behavior modification, physical therapy and oxygen-ozone therapy [1]. New treatment strategy such as radial Extracorporeal Shock Wave Therapy is effective in relieving pain and improve function in patients with myogenic TMDs [5]. The abbreviations used in the text is listed in Table 1.

**Table 1:** The list of abbreviations used in the text

Abbreviation	Definition
BMI	Body mass index
DC/TMD	Diagnostic Criteria for Temporomandibular Disorders
GWAS	Genome-wide association studies
IL-1B	Interleukin-1 Beta
IVs	Instrumental variables
IVW	Inverse-variance weighted
MMP-3	Matrix metalloproteinase-3
MR	Mendelian randomization
MR-PRESSO	Mendelian randomized polymorphism RESidual Sum and Outlier
RCTs	Randomized controlled trials
SNPs	Single nucleotide polymorphisms
TMDs	Temporomandibular disorders
TMJ	Temporomandibular joint

Risk factors consistently associated with TMDs include oral parafunctions, trauma, other pain conditions (e.g., chronic headaches), sleep apnea and psychiatric illness [1, 4]. Moreover, recent observational studies suggested certain systemic disorders or diseases (e.g., COVID-19 infection, disturbance of menstrual statuses and estrogen levels, diabetes, osteoarthritis) might play an important role in TMDs occurrence through promoting inflammation, disrupting bone metabolism balance in temporomandibular joint (TMJ) or exacerbating anxieties, amplifying the vulnerability of individuals [1, 6–8]. However, the causal effect and preventive significance of the associations between the systemic disorders and TMDs remains limited. More research is needed to identify other potential risk or protective factors for TMDs, such as obesity [2], in order to reduce the burden of this condition.

Obesity, calculated by body mass index (BMI), refers to an abnormal accumulation of adipose tissue within the whole body [9]. Adipose tissue is considered to be associated positively with inflammation and immunity [10, 11]. Several observational studies supported obesity as a risk factor for TMDs [2, 12–15]. In vivo, the obese mice fed high-fat diets presented with osteoarthritis-like changes and increased the expression of Interleukin-1 Beta (IL-1B), Matrix metalloproteinase-3 (MMP-3) and leptin in temporomandibular joints [13]. Moreover, the CC allele of rs8044769 in the FTO gene (an obesity-associated gene) is proved as a risk factor for temporomandibular joint osteoarthritis [16]. Conversely, patients with TMDs might have difficulty in food intake and tend to lose weight [17, 18]. According to the available research, the association between obesity and TMDs occurrence remains unclear due to conflicting findings [19–21]. The above studies are mainly based on a relatively small sample and likely to be affected by numerous potential confounding factors and prone to reverse causation. To date, no systematic examination of the association between obesity and TMDs has been conducted, and whether obesity plays a causal role in the development of TMDs remains unknown.

Mendelian randomization (MR) utilizes genetic variants, typically single nucleotide polymorphisms (SNPs), as instrumental variables (IVs) to infer the causal effect of an exposure variable on an outcome variable [22, 23]. When randomized controlled trials (RCTs) are not available, the MR approach provides a valuable alternative for estimating causal effects with great reliability. Like RCTs, genetic variants are randomly assigned at conception, thereby reducing the effect of confounders [23]. MR has emerged as a widely-used and effective method for investigating causal relationships using large-scale, publicly available data from genome-wide association studies (GWAS) and GWAS meta-analyses [24]. Recent MR studies suggest overweight or obesity as a risk factor for the joint diseases, such as knee, hip, and hand osteoarthritis or rheumatoid arthritis [22, 25], as well as bone health [26].

Therefore, this study aimed to investigate the direction and causal relationship between BMI and TMDs using a bidirectional MR approach, so as to provide a new perspective on the prevention and treatment of TMDs.

## Methods

### Study design

**Fig. 1:**
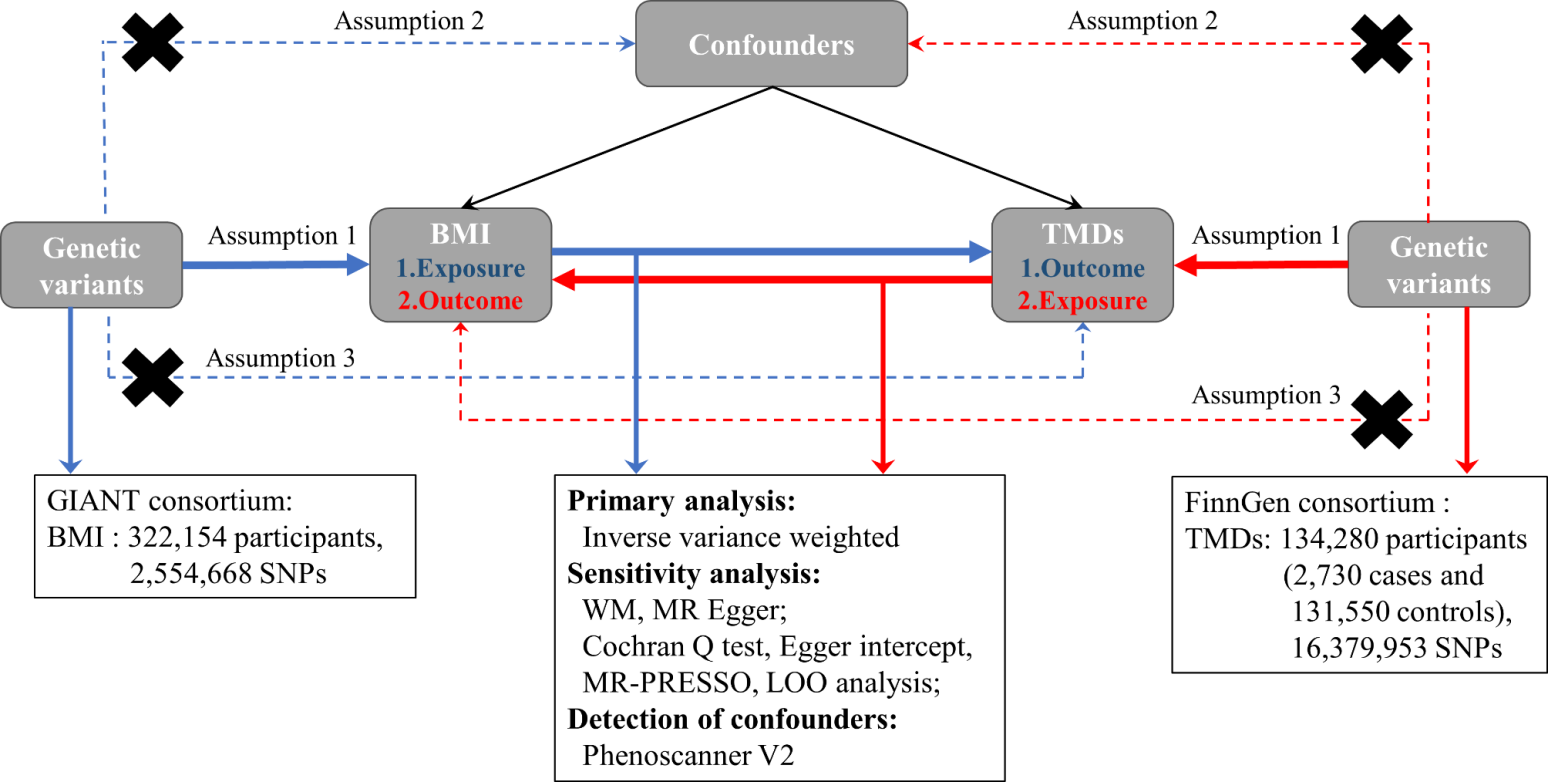
Overview of the present Mendelian randomization study. Assumption **1**, genetic variants are strong correlated with exposure; Assumption **2**, genetic variants are independent of confounding factors associated with exposure and outcome; Assumption **3**, genetic variants influence outcome only through the exposure; TMDs, temporomandibular disorders; BMI, body mass index; WM, weighted median; LOO, leave-one-out; SNPs, single nucleotide polymorphisms

An overview of the study design is presented in Fig. 1. The genetic variants should meet the following three assumptions. First, genetic variants are strongly correlated with exposure. Second, genetic variants are independent of confounding factors associated with exposure and outcome. Third, genetic variants influence outcome only through the exposure. The latter two assumptions, collectively referred to as the assumption of no horizontal pleiotropy, can be tested indirectly using various statistical methods [27]. The present MR study was based on the previously collected and published data, no ethics approval was required.

### Participants and data source

Publicly accessible summary statistics data for BMI were obtained from the IEU OpenGWAS project website (https://gwas.mrcieu.ac.uk). Temporomandibular joint disorders dataset came from the European samples FinnGen project (https://results.finngen.fi/en). TMDs cases are defined by K07.60, K07.61, K07.62 and K07.63 in the revised International Classification of Disease (ICD-10) code (https://risteys.finngen.fi/endpoints/TEMPOROMANDIB). Two thousand seven hundred and thirty cases of TMDs and 131,550 control cases, 322,154 participants of BMI were obtained from GWAS data for the study. All the above populations are of European origin to minimize potential bias due to demographic heterogeneity. No additional ethics statement or consent was required for the present study.

### Selection of genetic instruments

To meet the first assumption, we extracted SNPs robustly associated with the exposure (*p* < 5 × 10^− 8^). In addition, PLINK clumping method was adopted to exclude SNPs (r^2^ ≥ 0.001, clumping window ≤ 10,000 kb), only the SNP with the lowest *p*-value was retained. For the other two assumptions, we uploaded each selected SNP to the PhenoScanner V2 website (http://www.phenoscanner.medschl.cam.ac.uk) to eliminate those associated with potential confounders (pain or psychosocial conditions) and the outcome. If exposure-related SNPs were not available in the resultant GWAS, proxy SNPs significantly associated with the variant of interest were selected (r^2^ > 0.8). For those absent and no appropriated proxies were identified, we discharged them. Next, we harmonized remaining exposure and outcome SNPs and removed ambiguous and incompatible SNPs. At last, we calculated R^2^ and F-statistics as described before to estimate the strength of instrumental variables [28]. Generally, F-statistics > 10 was set as the threshold of strong IVs [29].

### Statistical analysis

Several complementary MR analysis methods, including inverse-variance weighted (IVW) method, the weighted median method and MR Egger regression method, were used to accurately examine causal relationship between BMI and TMDs. The IVW method was adopted as the major MR analysis. Sensitivity analysis were used to identify underlying pleiotropy and heterogeneity for MR estimates. The MR Egger intercept is an indicator for horizontal pleiotropy. Additionally, we performed Mendelian randomized polymorphism RESidual Sum and Outlier (MR-PRESSO) test to identify outlier SNPs that may have affected the causal estimates due to horizontal pleiotropy. If any outlier SNPs identified, we would remove them and restart the MR analysis. The Cochran Q test was calculated to examine the heterogeneity among different genetic variations. To identify the consistency of the results, leave-one-out analysis was conducted with each SNP removed. All MR analysis were conducted using R (version 4.3.0) through TwoSampleMR package (version 0.5.6) and MRPRESSO (version 1.0).

## Results

### Causal effects of BMI on TMDs

After initial screening, 69 independent SNPs related to BMI were identified (Supplementary Table 1). In the PhenoScanner search, we eliminated 12 SNPs associated with pain, nerve, anxiety, depression or tense [17] (Supplementary Table 2). One variant (rs17066856) missing in the outcome dataset was discharged. In the harmonization, four variants (rs1558902, rs17001654, rs4256980, rs9579083) were excluded for being palindromic with intermediate allele frequencies (Supplementary Table 3). F-statistics of genetic instruments for BMI ranged from 29.0 to 225.7, indicating sufficient instrument strength for MR analysis (Supplementary Table 1). At last, a total of 52 SNPs were remained with BMI as the exposure and TMDs as the outcome.

**Fig. 2:**
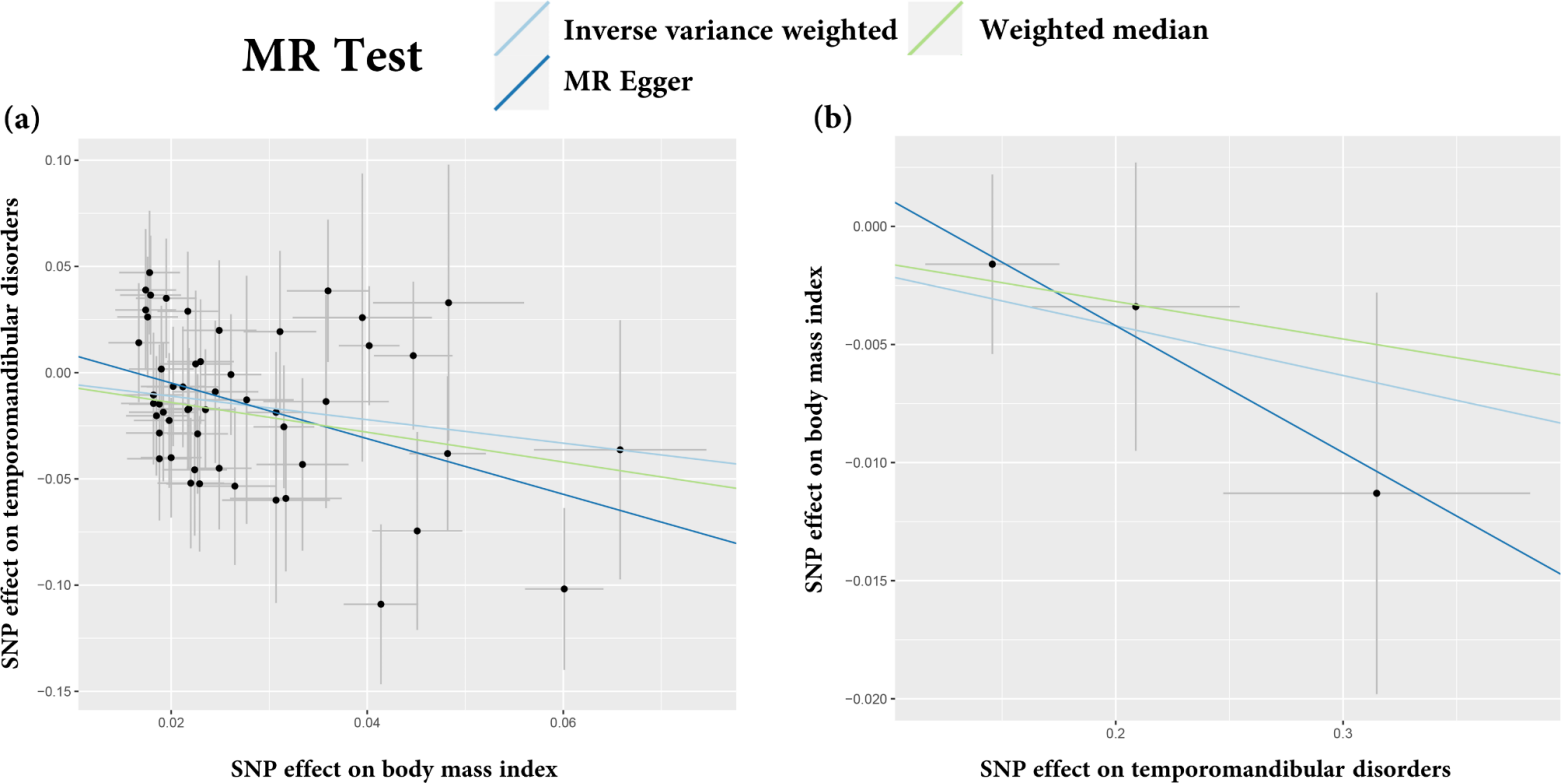
Scatter plot of the causal relationship between body mass index (BMI) and temporomandibular disorders (TMDs) using different MR methods. (**a**) Causal estimates for BMI on TMDs; (**b**) Causal estimates for TMDs on BMI.

**Fig. 3:**
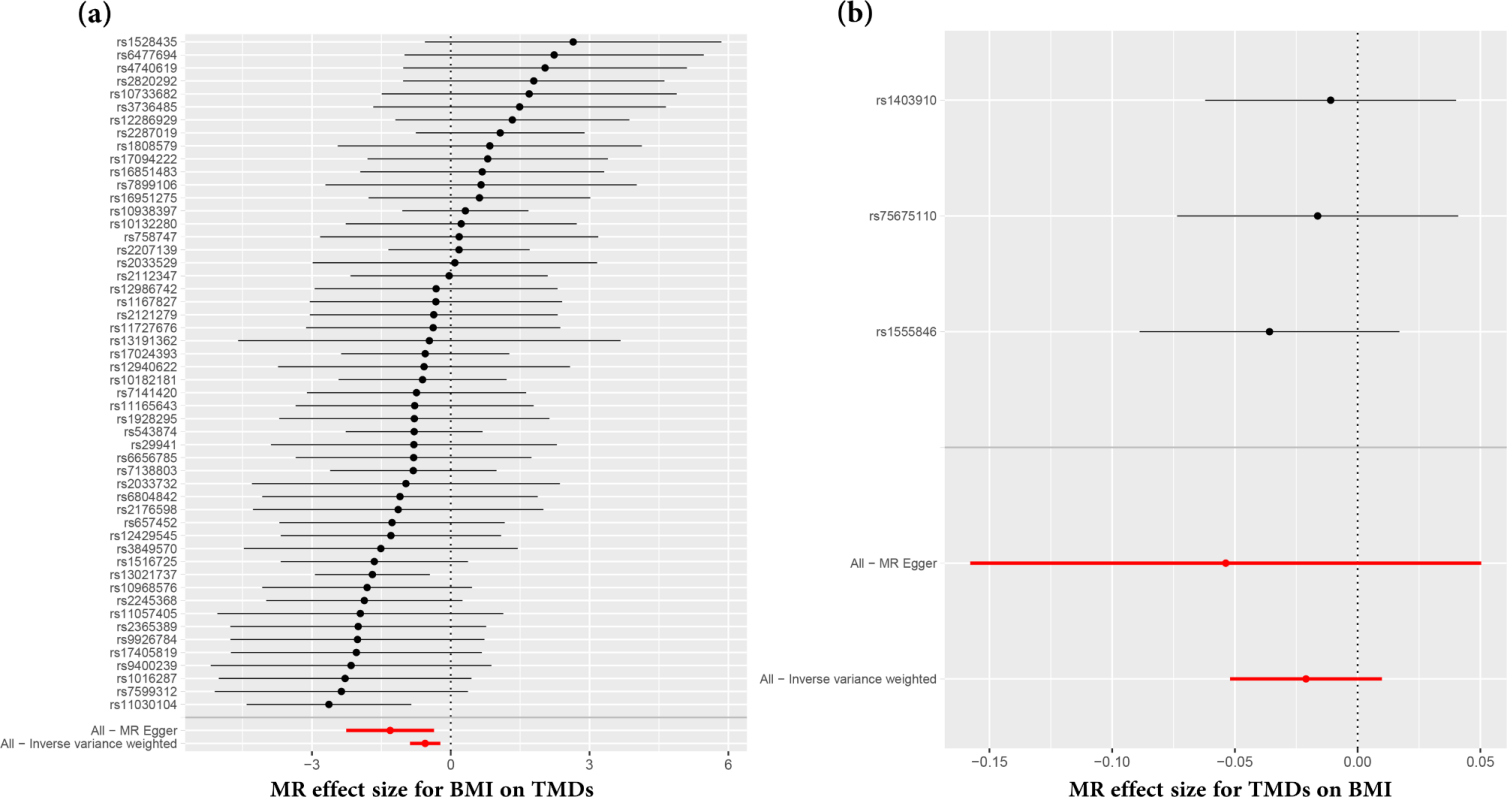
Forest plot of the causal effects of single nucleotide polymorphisms. (**a**) MR effect size for body mass index (BMI) on temporomandibular disorders (TMDs); (**b**) MR effect size for TMDs on BMI. The significance of red lines are MR results of MR Egger test and inverse variance weighted method

In the main IVW, genetically predicted BMI was significantly associated with a decreased risk of TMDs (OR = 0.575, 95% CI = 0.415–0.798, *p* = 0.001) (Table 2; Fig. 2a). MR Egger (OR = 0.270, 95% CI = 0.104–0.698, *p* = 0.009) and weighted median method (OR = 0.496, 95% CI = 0.298–0.826, *p* = 0.007) showed consistent results. Figure 3a displayed scatter plots of SNP effect sizes for BMI and TMDs. There was no evidence of heterogeneity (IVW Q = 50.700, I^2^ = 0.006, p = 0.485; MR Egger Q = 47.933, I^2^ = 0.043, *p* = 0.557) or pleiotropy (Egger intercept = 0.021, *p* = 0.102), as shown in Table 3. MR-PRESSO identified no outlier SNPs (*p* = 0.262). Additionally, leave-one-out analysis did not reveal any SNP that disproportionately influenced the overall effect of BMI on TMDs occurrence (Supplementary Fig. 1). The funnel plot displayed evidence of asymmetry (Supplementary Fig. 2), indicating the absence of pleiotropy.

**Table 2:** Estimated causal relationship between BMI and TMDs using different MR methods

Outcome	Exposure	Methods	N. SNPs	OR	95% CI	*P*
TMDs	BMI	IVW	52	0.575	0.415–0.798	0.001
		MR Egger	52	0.270	0.104–0.698	0.009
		Weighted median	52	0.496	0.298–0.826	0.007
Outcome	Exposure	Methods	N. SNPs	Beta	SE	*P*
BMI	TMDs	IVW	3	-0.021	0.016	0.182
		MR Egger	3	-0.054	0.053	0.496
		Weighted median	3	-0.016	0.020	0.422

Abbreviations: TMDs, temporomandibular disorders; BMI, body mass index; N. SNPs, number of SNPs used in MR; OR, odds ratio; IVW, inverse variance weighted

**Table 3:** Sensitivity analysis of the associations between BMI and TMDs.

Outcome	Exposure	Methods	Heterogeneity statistics			Horizontal pleiotropy	
			Q	*I* ^*2*^	*P*	Egger intercept	*P*
TMDs	BMI	IVW	50.700	0.006	0.485		
		MR Egger	47.933	0.043	0.557	0.021	0.102
BMI	TMDs	IVW	0.478	3.184	0.787		
		MR Egger	0.063	14.991	0.803	0.007	0.636

Abbreviations: TMDs, temporomandibular disorders; BMI, body mass index; IVW, inverse variance weighted

### Causal effects of TMDs on BMI

In reversed MR analysis, we adjusted the association threshold to 5 × 10^− 6^ because only a limited number of SNPs reaching previous genome-wide significance. Taking TMDs as exposure and BMI as outcome, a total of 3 SNPs (rs1403910, rs1555846, rs75675110) were finally extracted and F-statistic value ranged from 20.9 to 24.4 (Supplementary Table 4). The result of MR indicated that TMDs had no causality on BMI (all *p* > 0.05) (Table 2; Fig. 2b). Scatter plots of SNP effect sizes for TMDs and BMI are shown in Fig. 3b. Egger intercept showed little evidence of horizontal pleiotropy (Egger intercept = 0.007, p = 0.636). The Cochran Q-test yielded *p*-values greater than 0.787, indicating a lack of heterogeneity among the included SNPs (Table 3). Leave-one-out analysis was conducted by removing individual SNP from instruments in turn, and the estimated effects derived from the IVW model remained null (Supplementary Fig. 3).

## Discussions

To date, this is the first study to explore the bidirectional causal relationship between BMI and TMDs by performing multiple complementary MR approaches. Interestingly, forward MR analysis showed evidence that BMI were causally associated with TMDs, and lower BMI increased the risk of TMDs. No evidence of BMI and TMDs associations supporting genetic prediction was detected in our two-sample MR reverse analysis.

A body of studies have investigated whether there is a link between BMI and TMDs in recent years. One proposed theory suggests that obesity-induced chronic inflammatory factors may participate in the pathological process of joint diseases. Leptin is one of the increased proinflammatory factors in obese people. In addition, excessive leptin expression was observed in the condylar chondrocytes under inflammation in vitro. Several cross-sectional studies have reported a positive association between BMI and the severity of TMDs [14, 15]. A recent study used artificial intelligence methodologies on a large sample of 4744 participants to classify the etiological factors of TMDs. When analyzing both self-reported and doctor-diagnosed TMDs data, BMI was one of the most important positive factors [2]. In a twin study, overweight individuals were found to have a higher incidence of TMDs or muscular disorders [12]. However, these results may be limited by several reasons, such as different diagnose methods, insufficient samples, a small study span and limited portion of TMDs. It is worth noting that the age range and gender distribution of the study population may limit the generalizability of these findings, as TMDs are more prevalent among females between the ages of 20 and 40 [17].

To data, the largest related cross-section study was conducted based on the Korea National Health and Nutrition Examination Survey. A total of 119, 222 adults were included and performed TMDs examination following standardized criteria. The subsequent analysis showed subjects with TMDs tended to have a decreased prevalence of obesity. Univariate and multivariate logistic regression analyses further suggested low BMI as a risk factor for TMDs [19]. This study is more convincing as its large sample size and the standardized diagnosis of TMDs by dentists. Moreover, this survey considered different age groups including those prone to TMDs. Other studies have also reported consistent findings [30, 31]. Our MR analysis provides additional evidence supporting a causal relationship between low BMI and an increased risk of TMDs. The negative association between BMI and TMDs is biologically plausible. First, the altered sex hormonal metabolism in obese individuals may be a protect factor for TMDs. Estrogen could accelerate bone remolding by promoting stemness and osteogenesis, and inhibiting senescence of bone marrow stromal cells through an ERB-SATB2 pathway [32]. In addition, estrogen possesses a protective factor on the TMJ chondrocyte through inhibiting the expression of nitric oxide [33]. However, estrogen was in a lower level of underweight female adolescents [33, 34], indicating that those with low BMI might not have strong ability for TMJ repairment or remolding. Testosterone plays a key role in enhancing muscle mass and strength. In obese men, testosterone could be readily converted into estradiol via aromatization in adipose tissue [35]. Therefore, the temporomandibular joint may be protected by decreasing masticatory muscle strength and bite force in obese men through testosterone conversion. Additionally, dietary habits may affect the prevalence of TMDs by altering mechanical stress on the TMJ [1]. Eating behavior such as preferring soft fatty food, avoiding hard food and chewing less are common in obese individuals. While a higher intake of fiber is more often seen in underweight adults [19].

Patients with TMDs might have trouble in food intake and chewing due to orofacial pain and limited range of jaw movement [36]. Large-scale observational studies have reported no significant association between TMDs and changes in BMI over time among the general population [37, 38]. Our reverse MR analysis did not provide any evidence supporting a causal effect of TMDs on BMI changes. The modified diet, including cutting food in to smaller pieces, softening and mashing food, might prevent nutritional deficits in subjects with TMDs [38]. It is important to note that the limited number of SNPs available for extraction when using TMDs as the exposure may result in some degree of information loss and selection bias.

Previous observational studies reporting on the association between BMI and TMDs may have been affected by unmeasured confounding factors, as well as bidirectional causation [2, 19]. Our MR research support the possibility that BMI and TMDs are causal. The IVW method is designed to assess the causal relationship by the meta-analysis of each Wald ratio for the selected SNPs [39]. Specifically, IVW assumes that all the genetic variants are valid, and thus is the most powerful tool for MR estimation but also prone to pleiotropic bias [27]. Weighted median permits a consistent estimate accounting for pleiotropy when half of the instruments are invalid, while MR-Egger allows for the analysis when encountering all invalid instruments [40]. Horizontal pleiotropy occurs when genetic variants related with the exposure of interest directly affect the outcome through multiple pathways [41]. Fortunately, no evidence of horizontal pleiotropy and heterogeneity were identified using complementary statistical methods in the MR study. All MR approaches led to consistent results, indicating the robustness of the present findings.

A multidisciplinary approach is successful for the management of TMDs [1]. The findings from our study might provide new evidence and strategies for preventing and treating TMDs. Dentists should consider screening patients with low BMI for signs and symptoms of TMDs, such as jaw pain, clicking or popping sounds when opening or closing the mouth, difficulty chewing, and headaches. Early identification and intervention can help prevent the progression of TMDs and alleviate symptoms [1]. Moreover, the importance of weight control should be emphasized in patient education and treatment plans for individuals with TMDs. Clinicians may recommend weight management interventions such as dietary modifications and lifestyle changes. However, a recent study of orthodontists showed that 55% never collect any weight information and 73% did not assess for obesity in any way [42]. Dentists should pay greater attention to weight records and assessment of patients, especially for those with low BMI who seek medical advice for TMD-related discomfort.

The findings of the current study should be interpreted with caution. The limitations of the research are introduced as follows. First, this survey was conducted in a European population. It might be hard to generalize the results as causality may depend on ethnicity and selection bias. Further MR studies should be conducted in diverse populations. Second, despite our efforts to account for pleiotropy using multiple approaches, it is possible that some residual pleiotropic effects may still exist. Third, our study relied solely on BMI to assess obesity risk, but variations in adipose tissue distribution could also influence the results. Forth, we initially made efforts to identify age and gender-stratified GWAS data because factors including age and sex played an important role in both TMDs and BMI [17, 43]. However, no such BMI and TMDs datasets could be found. We then had to adopt GWAS data which was adjusted for age and sex [44]. Thus, it is not able to answer whether BMI during one particular life cycle has any effect on the occurrence of TMDs in different genders. Note that almost all GWAS have limitations in directly providing information on comorbidity. Fifth, although that the MR approach performs excellently in causal estimates, validation of our findings through additional RCTs would help establish causality.

## Conclusion

This study indicated that low BMI might increase the risk of TMDs in a causal way, supporting the importance of weight control for the prevention and treatment of TMDs. Clinicians should pay more attention to the low-BMI patients among those seeking medical advice for TMD discomfort.

## Electronic supplementary material

Below is the link to the electronic supplementary material.

Supplementary Material 1

Supplementary Material 2

Supplementary Material 3

Supplementary Material 4

Supplementary Material 5

## Data Availability

All data generated or analyzed during this study are included in supplementary material or in the data repositories listed in the methods.
