# Supplementary material for: Causal association between body mass index and temporomandibular disorders: a bidirectional two-sample Mendelian randomization analysis
Source: BMC Oral Health. 2023 Jul 18;23:499. doi: 10.1186/s12903-023-03179-5 (PMC10355070; doi:10.1186/s12903-023-03179-5)
Supplement: Supplementary file 1 — Supplementary Material 1 [file 12903_2023_3179_MOESM1_ESM.docx]

Supplementary Fig 1. Leave-one-out meta-analysis. The leave-one-out plot visualized how the causal estimates (point with horizontal line) for the effect of body mass index (BMI) on temporomandibular disorders (TMDs) were influenced by the removal of single variant.
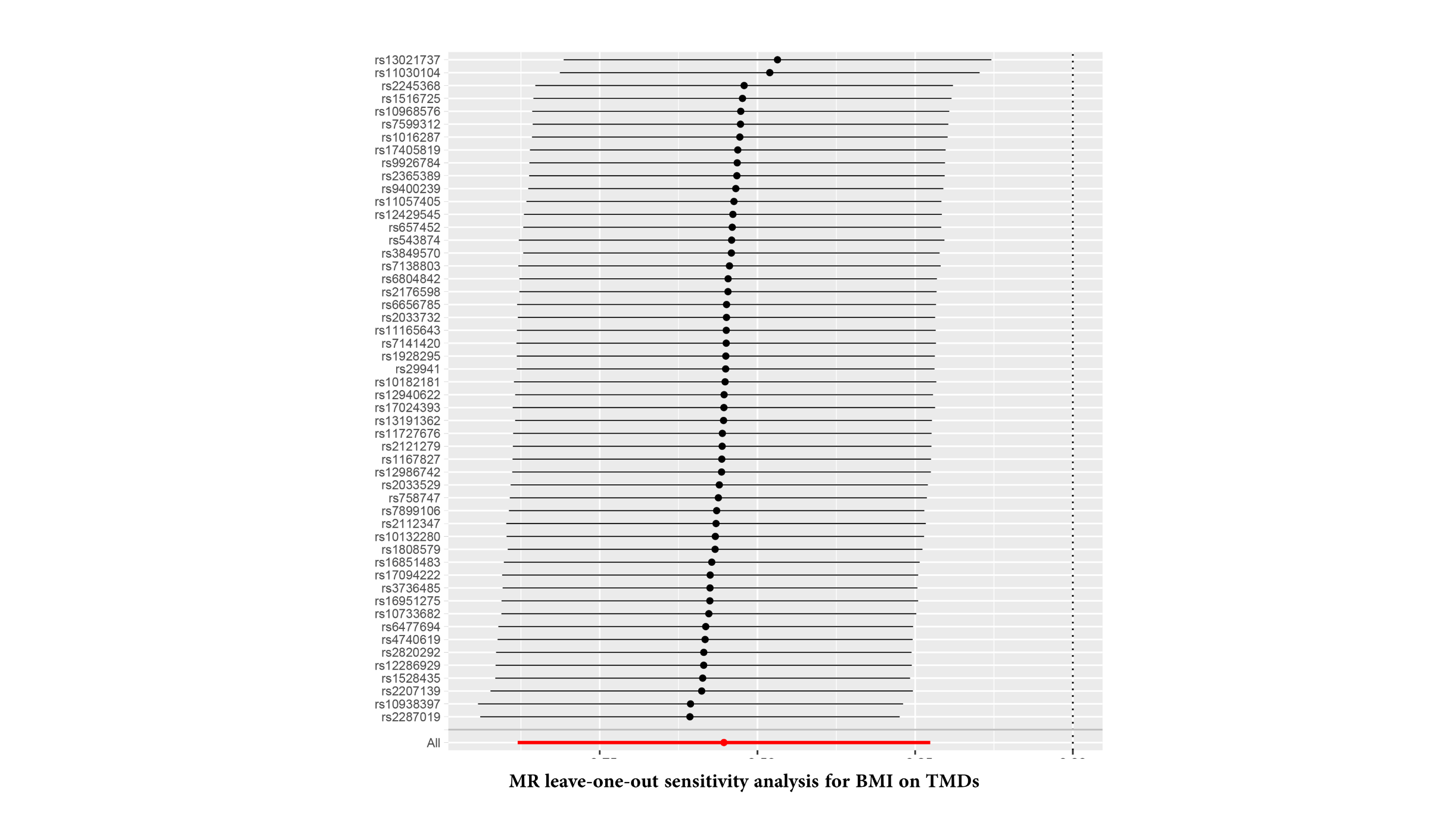


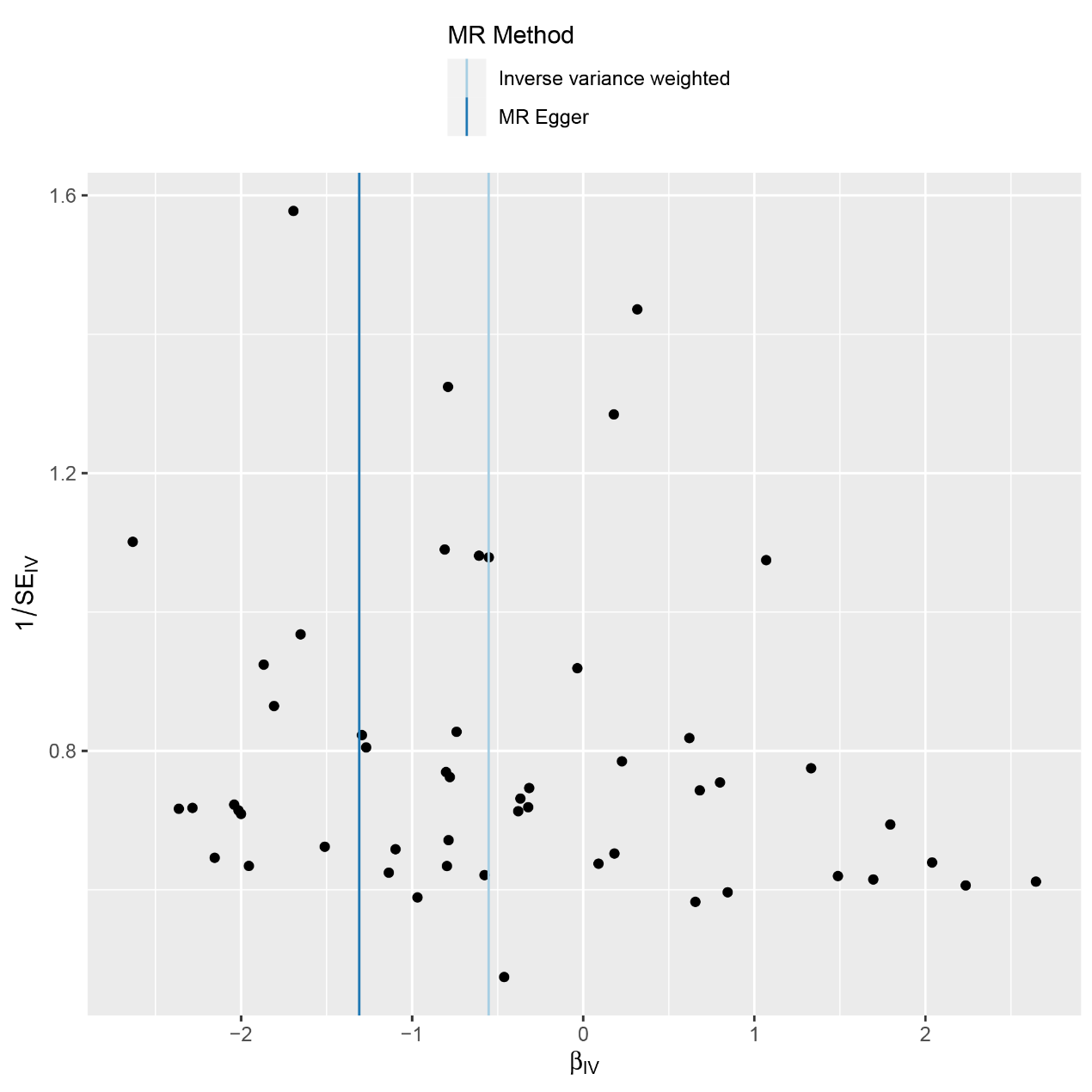


Supplementary Fig 2. Funnel plot of SNPs associated with body mass index and their risk of temporomandibular disorders.


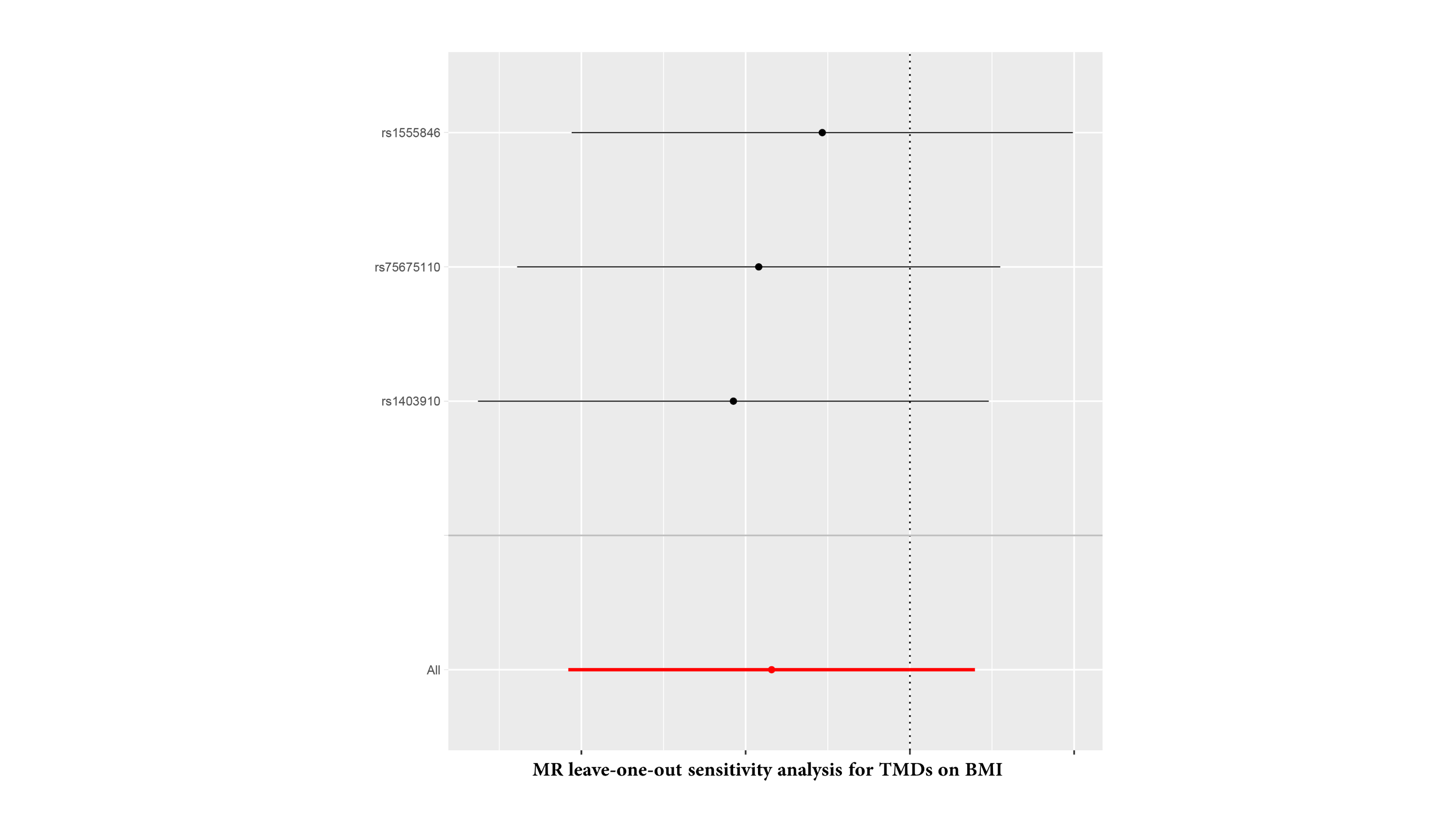


Supplementary Fig 3. Leave-one-out meta-analysis. The leave-one-out plot visualized how the causal estimates (point with horizontal line) for the effect of temporomandibular disorders (TMDs) on body mass index (BMI) were influenced by the removal of single variant.
